# The RNA-Binding Protein, Polypyrimidine Tract-Binding Protein 1 (PTBP1) Is a Key Regulator of CD4 T Cell Activation

**DOI:** 10.1371/journal.pone.0158708

**Published:** 2016-08-11

**Authors:** James La Porta, Rodrigo Matus-Nicodemos, Aníbal Valentín-Acevedo, Lori R. Covey

**Affiliations:** Department of Cell Biology and Neuroscience, Rutgers University, New Brunswick, New Jersey, United States of America; University of Miami, UNITED STATES

## Abstract

We have previously shown that the RNA binding protein, polypyrimidine tract-binding protein (PTBP1) plays a critical role in regulating the expression of CD40L in activated CD4 T cells. This is achieved mechanistically through message stabilization at late times of activation as well as by altered distribution of CD40L mRNA within distinct cellular compartments. PTBP1 has been implicated in many different processes, however whether PTBP1 plays a broader role in CD4 T cell activation is not known. To examine this question, experiments were designed to introduce shRNA into primary human CD4 T cells to achieve decreased, but not complete ablation of PTBP1 expression. Analyses of shPTB-expressing CD4 T cells revealed multiple processes including cell proliferation, activation-induced cell death and expression of activation markers and cytokines that were regulated in part by PTBP1 expression. Although there was an overall decrease in the steady-state level of several activation genes, only IL-2 and CD40L appeared to be regulated by PTBP1 at the level of RNA decay suggesting that PTBP1 is critical at different regulatory steps of expression that is gene-specific. Importantly, even though the IL-2 protein levels were reduced in cells with lowered PTBP1, the steady-state level of IL-2 mRNA was significantly higher in these cells suggesting a block at the translational level. Evaluation of T cell activation in shPTB-expressing T cells revealed that PTBP1 was linked primarily to the activation of the PLCγ1/ERK1/2 and the NF-κB pathways. Overall, our results reveal the importance of this critical RNA binding protein in multiple steps of T cell activation.

## Introduction

Over the past two decades it has become increasingly clear that posttranscriptional events are critical for appropriate cellular responses in both innate and adaptive immunity. These processes come into play subsequent to transcription, splicing, and the capping of precursor transcripts, and orchestrate the integration of cellular activities including nuclear export, cytoplasmic localization, translation initiation and mRNA decay [1–3]. Lymphocyte activation presents a unique challenge for integrating transcriptional and posttranscriptional processes because of the requirement for cells to immediately respond to environmental cues by undergoing rapid phenotypic and functional changes. These dramatic shifts in gene expression rely not only on transcription but also on regulated mRNA decay to fine tune the level of a particular transcript at any given time during the activation cycle. Regulated mRNA decay can come about by RNA binding proteins (RBPs), microRNAs or both acting together on the same transcript (reviewed in [4]).

Our work over the past several years has focused on understanding molecular signals that regulate critical “helper” properties of CD4 T cells that provide non-redundant differentiation and activation signals required to B cells and other antigen-presenting cells (APCs) (reviewed in [5]). In particular, work has focused on understanding posttranscriptional mechanisms that regulate the expression of CD40 ligand (CD40L), a member of the TNF superfamily of genes expressed primarily on activated CD4 T cells, basophils, mast cells and platelets, and is required for both class switch recombination and somatic hypermutation in antigen-selected B cells (reviewed in [5]). Expression of CD40L is controlled at multiple levels by transcriptional, posttranscriptional and translational mechanisms [6–10]. Additionally, CD40L is removed from the cell surface following engagement with CD40 underscoring the importance of limiting bystander cell activation by CD40L-expressing T cells [11]. At the posttranscriptional level, CD40L mRNA turnover is governed by an activation-dependent mechanism that leads to the rapid degradation of transcripts up to 8 h following CD3 or CD3 plus CD28 stimulation with a half-life or *t*_1/2_< 35 min and significant stabilization of the transcript at late times of activation [12]. This program of “regulated stability” is highly distinct from other TNF family members and is mediated by the binding of an RNA complex that is dependent both on T cell activation as well as on the presence of an RBP termed polypyrimidine tract-binding protein 1 or PTBP1. This protein forms a complex with additional RBPs and binds to a region in the 3’ untranslated region (UTR) of the CD40L message at late times of activation leading to its stabilization [13–16].

In addition to a role in mRNA stability for a number of known transcripts [17, 18], PTBP1 is critical at multiple steps of RNA biogenesis including 3′-end cleavage, polyadenylation, mRNA export from the nucleus, and viral and cellular gene IRES-mediated translation [19–21]. For example, we have found that PTBP1 is critical for distributing CD40L mRNA between the nuclear and the cytoplasmic compartments [22]. However, PTBP1 has been most thoroughly studied as a critical factor in alternative intronic splicing of viral and cellular RNAs where it acts to block inclusion of alternative exons into mRNA [19, 23, 24]. Furthermore, it has been shown that PTBP1 together with heterogeneous nuclear ribonucleoprotein A1/2 slicing factors are critical for the generation of pyruvate kinase M2 (PKM2) required for aerobic glycolysis in proliferating tumor cells [25].

The identification of a PTBP1-mediated pathway of CD40L regulation provided an opportunity to identify novel factors that govern CD4 helper function at specific points of activation. Since CD40L is often deregulated in autoimmune disease and cancer, defining pathways that are critical for optimal expression under normal conditions will undoubtedly uncover new approaches for therapeutic intervention [26–28]. However, to utilize this pathway for regulating T helper function, a more global understanding of the impact that PTBP1 has on T cell activation is essential. Therefore, in this study we have asked questions regarding the ability of PTBP1 to affect a number of different CD4 T cell responses by using primary human cells in which PTBP1 is downregulated using shRNA-specific gene targeting. Our results show that PTBP1 is critical for optimal proliferation, survival and expression of several activation markers including CD25 and IL-2 and that PTBP1-mediated control occurs at multiple levels of gene regulation. Furthermore, we identify the ERK1/2 and NFκB pathways as major targets of PTBP1 at early time points following activation.

## Materials and Methods

### Ethics Statement

De-identified human blood was obtained from the blood bank of the Affiliated Hospitals of Rutgers University, Robert Wood Johnson Medical School. Protocol (E13-414) was submitted to the Rutgers University IRB committee and notice of exemption category 4 was approved on 1/9/13.

### Antibodies

The anti-human-PTBP1 mAb BB7 (ATCC number: CRL-2501) was purified as previously described [29]. Streptavidin- Phycoerythrin (PE) and biotin-conjugated anti-CD69 (clone FN50), anti-CD40L (clone BMS153BT), CD25 (clone BC96), PE-conjugated anti-IFNγ (clone 45B3), -TNFα (clone 45B3), -IL-2 (clone MQ1-17H12), and -CD4 (clone RPA-T4) were purchased from eBioscience. The biotin labeled anti-human CD38 (clone AT1) was purchased from Ancell. Phospho-specific anti-PKCθ (T538),—PLCγ1 (Tyr783), and appropriate isotype controls, as well as rabbit polyclonal anti-PLCγ1 and anti-ERK1/2 (clone 9102) were purchased from Cell Signaling. Phospho-specific antibodies against NFκB (S529), -p38MAPK (pT180/pY182), ERK1/2 (pT202/pY204) and PE-conjugated mouse anti-human IκBα (clone 25) and appropriate isotype controls were purchased from BD-Biosciences. Phospho-specific anti-STAT5 (Y694) (clone SRBCZX) was purchased from Affymetrix.

### Lentiviral gene transduction

The engineering of the pLVTHM-U6-CTRL (pLV-CTRL) and pLVTHM-U6-shPTB (pLV-PTB) constructs together with the protocol for lentiviral production have been previously described [22, 30]. Briefly, 5X10^5^ 293T cells were plated into 6-well plates with 3 ml of DMEM media supplemented with 1% heat-inactivated FBS, 50 U/ml penicillin, 50 μg/ml streptomycin, 1% NaPyruvate, 1% minimal non-essential amino acids and 1 mM L-glutamine (DMEM-complete-1%). One day after plating, cells were transfected with 1.0 μg pLV-PTB or pLV-CTRL vectors with 0.8 μg psPAX2 (Addgene plasmid 12260) and 0.2 μg of pCI-VSVG (Addgene plasmid 1733) using Fugene HD (Promega) according to the manufacture’s protocol. Supernatants were collected at 24 and 48 h and concentrated by centrifugation at 25K for 1.5 h at 4°C. Virus was resuspended in 400 μl RPMI 1640 supplemented with 10% fetal bovine serum (FBS), 50 U/ml penicillin, 50 μg/ml streptomycin, 1% L-glutamine, 1% sodium pyruvate and 1× non-essential amino acids) (RPMI-complete) and left o/n at 4°C and was either frozen at -80°C or used immediately. Infection was carried out at approximately 20 PFU/cell.

PBMCs were isolated from blood drawn from healthy volunteers and obtained from the blood bank of the Affiliated Hospitals of Rutgers University, Robert Wood Johnson Medical School. CD4 T cells were isolated by negative selection using Miltenyi beads and 1-3X10^6^ CD4 T cells were transduced with viral supernatant expressing either shPTB or shCTRL in RPMI-complete in the presence of 100 U/ml rIL-2 and 0.1 μg/ml PHA (Calbiochem) or human T cell activator CD3/CD28 beads (Gibco) (5 cells/bead). Infection efficiency was between 20–48% of the CD4pos T cells. Cells were expanded with 100 U/ml rIL-2 at day 3 and subsequently 50 U/ml every other day. At 10–13 d post infection, the cells were placed into RPMI-complete without rIL-2 and rested for 1 day. Cells were activated with 1 ng/ml PMA and 1 μg/ml ionomycin for 5 h or with Dynabeads human T cell activator CD3/CD28 beads (Gibco) for 2 h and 48 h.

In experiments requiring sorted, uninfected and infected CD4 T cells, negatively selected CD4 T cells were infected and at day 2 post-infection sorted using a Coulter MoFlo XDP Cell Sorter into GFPneg and GFPpos populations. Sorted populations were expanded as described above and stimulated with anti-CD3/anti-CD28 mAbs-coated beads for 2 h and 48 h.

### Cell death and proliferation

#### Annexin V staining

Lentiviral vector-transduced CD4 T cells (1X10^5^) were harvested prior to or after 48 h activation with anti-CD3 + anti-CD28 mAbs-coated beads and incubated with PE-conjugated Annexin V for 30 min according to the manufacturer’s protocol (BD Biosciences). The stained cells were analyzed immediately by flow cytometry analysis using a FACSCalibur (Becton Dickinson, Mountain View, CA).

#### Proliferation studies

2x10^6^ CD4 T cells from pLV-PTB or pLV-CTRL -infected cultures were washed 2X and resuspended in 1mL of PBS. eFluor^®^ 670 (eBioscience) (5μM) was added and the cells vortexed and incubated at 37°C in the dark for 10 min. Labeling was quenched with the addition of 10mL cold RPMI-complete. Samples were washed 3X in RPMI-complete and 5x10^5^ cells collected and fixed as Day 0 samples. Remaining cells were plated and cultured, with 5x10^5^ cells being collected and fixed each subsequent day for 2 or 3 days. Data was collected and analyzed using FloJo software.

### Analysis of surface expression of activation markers

Cells were collected, washed and resuspended in 100 μl FACS wash buffer (1X PBS, 3% FBS, 0.1% NaN_3_,). Cells were incubated with 5 μg heat-aggregated IgG for 10 min on ice, washed with 3 ml FACS wash and resuspended in 100 μl FACS wash. IgG -coated cells were incubated for 45 min at 4°C with saturating amounts of biotin-conjugated mAbs or the matched isotype controls. Directly conjugated or purified antibodies were added to the cells (at pre-determined concentrations) and incubated for 45 min at RT with shaking. Alternatively, some cells were stained with antibodies that were directly conjugated to PE or APC. Cells were washed in FACS wash buffer, and biotin-conjugated samples were further incubated with PE-conjugated streptavidin for 30 min at 4°C. Cells were washed, fixed with 1% paraformaldehyde in FACS wash buffer and analyzed by flow cytometry.

### Intracellular staining

Primary CD4 T cell populations were cultured in 100 μl of media minus serum under activating conditions and fixed by adding 16% paraformaldehyde to a final concentration of 1.9%. After 10 min of incubation at RT, ice-cold MeOH (0.5 mL) was added to the cells, incubated at -20°C for 2 h to overnight, washed and resuspended in 100 μl of FACS wash buffer. Directly conjugated antibodies or unlabeled antibodies were added and cells incubated at 45 min at RT. Cells were washed with 3 ml of FACS wash and either resuspended in 1% paraformaldehyde/PBS (FACS fix) or stained with a labeled secondary antibody for 30 min at RT and analyzed by flow cytometry. For production of cytokines, cells were activated with 1 ng/ml PMA and 1 μg/ml ionomycin in the presence of 10 μg/ml brefeldin A (Sigma) for 5 h. Cells were fixed and permeabilized as above and subjected to intracellular staining with conjugated monoclonal antibodies.

### Steady-state gene expression and RNA decay analyses

RNA stability was assessed by incubating between 5x10^6^-1x10^7^ cells with 50 μg/ml of the transcription inhibitor 5,6-Dichlorobenzimidizole 1-β-D-ribofuranoside (DRB) (Sigma Chemical Co., St. Louis, MO). 1.25–2.5x10^6^ cells were collected at 0, 15, 30 and 60 min, pelleted by centrifugation at 400 x g and total RNA isolated using Trizol (Life Technologies). RNA was reverse transcribed with the Transcriptor First Strand cDNA Kit (Roche) as directed by the manufacturer using an anchored-oligo (dT) 23 primer. Real time quantitative PCR (qPCR) was performed using a StepOne Real Time PCR machine (Applied Biosystems) using buffer and conditions suggested by the manufacturer and primer sequences listed in S1 Table. Expression of individual genes was calculated using 18S [31] and/or β-actin as internal controls and fold differences determined by the ΔΔCT method.

### Immunoprecipitation of PTB-bound transcripts

To analyze the transcripts bound by PTBP1, 1 X 10^7^ purified CD4 T cells were cultured on 10-cc plates pre-coated with anti-CD3 mAb (clone HIT3α) and 4 μg/ml of added soluble anti-CD28 mAb (clone 9.35) for 48 h. Cytoplasmic extracts were prepared by resuspending cells in 150 μl cytoplasmic buffer (10 mM HEPES (pH 7.9), 10 mM KCl, 0.1 mM EDTA (pH 8.0) 0.1mM EGTA) with 1mM DTT, 1 mM sodium orthovanadate, 5 mM NaFl, 1 mM PMSF, and 1X Protease inhibitor Cocktail (Sigma). Extracts were incubated overnight at 4°C with agarose-A/G beads bound to anti-PTB Abs in NT2 buffer (50 mM Tris [pH 7.4], 150 mM NaCl, 1 mM MgCl_2_, 0.05% Nonidet P-40 and 40 U RNase inhibitor (RNAseOUT, Invitrogen)). The beads were washed six times with NT2 buffer and resuspended in 100 μl NT2 buffer with 0.1% SDS and 30 μg proteinase K, followed by incubation at 55°C for 3 h. RNA was extracted from the supernatant, reverse transcribed, and the cDNA amplified by real time PCR.

### Statistical Analyses

Statistical analyses were carried out using GraphPad Prism (version 6.0). An unpaired, two-tailed Student t test was applied for comparison of two groups. ANOVA was used with Bonferroni correction for multiple comparisons. A p value of ≤0.05 was considered to be significant.

## Results

### Optimal T cell proliferation requires PTBP1

**Fig 1:**
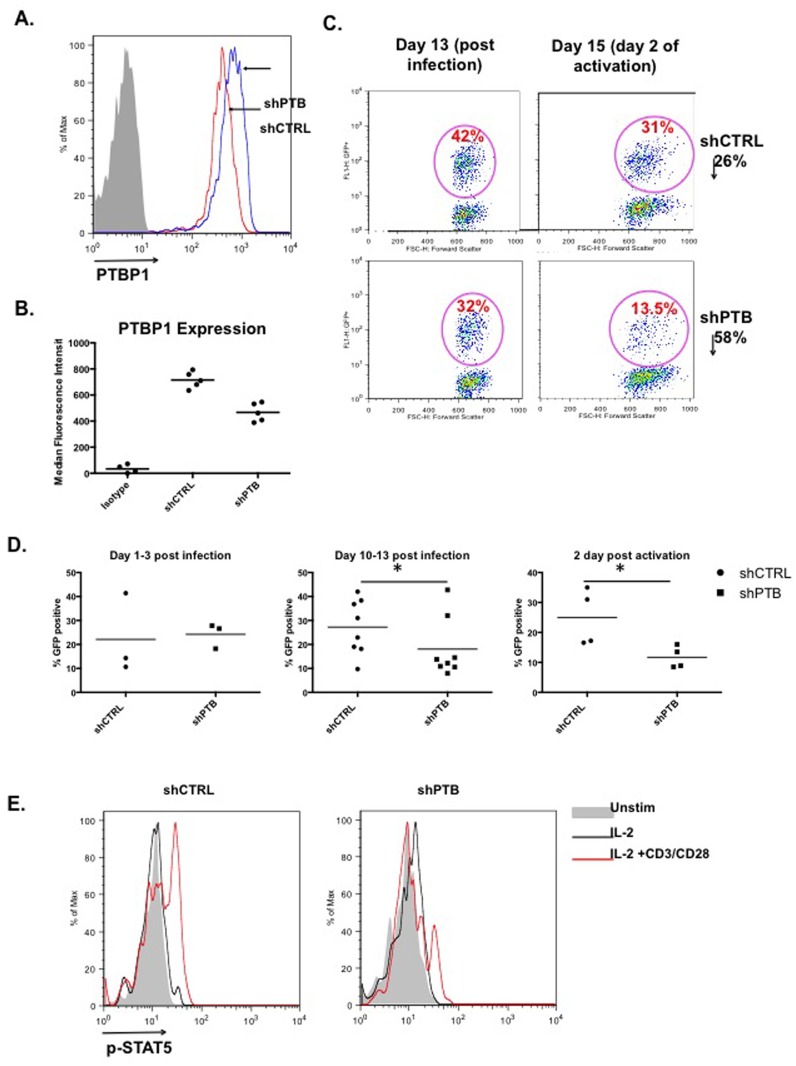
PTBP1 is required for optimal expansion of CD4 T cells. (A) Negatively selected CD4 T cells were infected with either pLV-shCTRL or pLV-shPTB lentivirus, expanded for 10 days in IL-2 and removed from IL-2 for one day. At day 11, GFPposCD4pos cells were analyzed for PTBP1 expression using intracellular immunostaining with anti-PTBP1 mAb or isotype control. Median fluorescence intensity (MFI) of 5 independent experiments is indicated in table below. (B) CD4 T cells infected with either pLV-shCTRL or pLV-shPTB were expanded for 13 days and activated for two days with anti-CD3 + anti-CD28 antibodies. The GFPpos population in each panel is presented as a percentage of the total CD4pos population. (C) Experiments were carried out as in “B” and data compiled from a minimum of three independent experiments. Comparisons between pLV-shCTRL or pLV-shPTB infected cultures were carried out at 1–3 and 10–13 days post infection and again 2 days post activation. (D) Analysis of phospho-STAT5 after a 10 day IL-2-dependent expansion, followed by either 2 days with no IL-2 (grey bar), or 1 day with no IL-2 and either a 20 min stimulation with 250 U of IL-2 (black line) or 250 U IL-2 plus anti-CD3 + anti-CD28 beads (red line).

To understand how PTBP1 broadly regulates T cell activation, primary human CD4 T cells were isolated and transduced with retroviral constructs expressing short hairpin (sh)RNAs against PTBP1 (shPTB) and a scrambled control (shCTRL) (pLV-shPTB and pLV-CTRL, respectively). Previously reported studies in which PTBP1 was completely ablated resulted in embryonic lethality due to defective organ development that was most likely linked to reduced cell proliferation [32–34]. Therefore, to circumvent this outcome shRNA hairpins were used that reduced rather than eliminated PTBP1 expression to approximately 40% of wild type levels (Fig 1A and 1B). Following activation and infection of CD4 T cells, cultures were maintained with the addition of only rIL-2 for 10–13 days to bring the activation state of the T cells close to baseline. Cells were then activated with antibodies to CD3 and CD28 or with PMA/Io. To insure that PTBP1 knockdown did not affect the expression of CD3 and CD28, which were engaged to activate CD4 T cells, cells were stained, analyzed by flow cytometry and shown to express comparable levels of both proteins in GFP-expressing cells (S1 Fig).

A comparison of infection profiles of infected CD4 T cells at the time of activation (10–13 days post-infection) frequently showed that the percent infection of shCTRL- expressing cells was markedly higher than shPTB-expressing cells (Fig 1C). To assess whether this difference represented fewer transduced cells or a loss of shPTB-expressing T cells in the expansion phase, infection was monitored at days 1–3 post infection and at day 10–13 following expansion. Cultures that were stimulated for 2 days with anti-CD3 and anti-CD28 were further analyzed for loss of cells following activation. Comparison of the GFPposCD4pos cells at early times of infection revealed no significant difference in the percentage of T cells infected with either shCTRL- or shPTB-expressing virus (Fig 1D, left panel). However, after IL-2-dependent expansion and again following anti-CD3/anti-CD28 activation there was a marked decrease in T cells in the shPTB pool (Fig 1D, middle and right panels). Because IL-2-dependent T cell proliferation requires the activation of STAT5 through phosphorylation of Y694, STAT5 was monitored for phosphorylation in response to either IL-2 or IL-2 plus anti-CD3 plus anti-CD28 antibodies for 20 min in control and cells expressing shPTB. We found reduced STAT5 phosphorylation in cells with lowered PTBP1 compared to activated control T cells (Fig 1E). Together these data suggest that wild type PTBP1 levels are critical for optimal expansion of CD4 T cells and that this dependence is linked to either loss of proliferative capacity and/or enhanced cell death.

### PTB is required for cell proliferation

**Fig 2:**
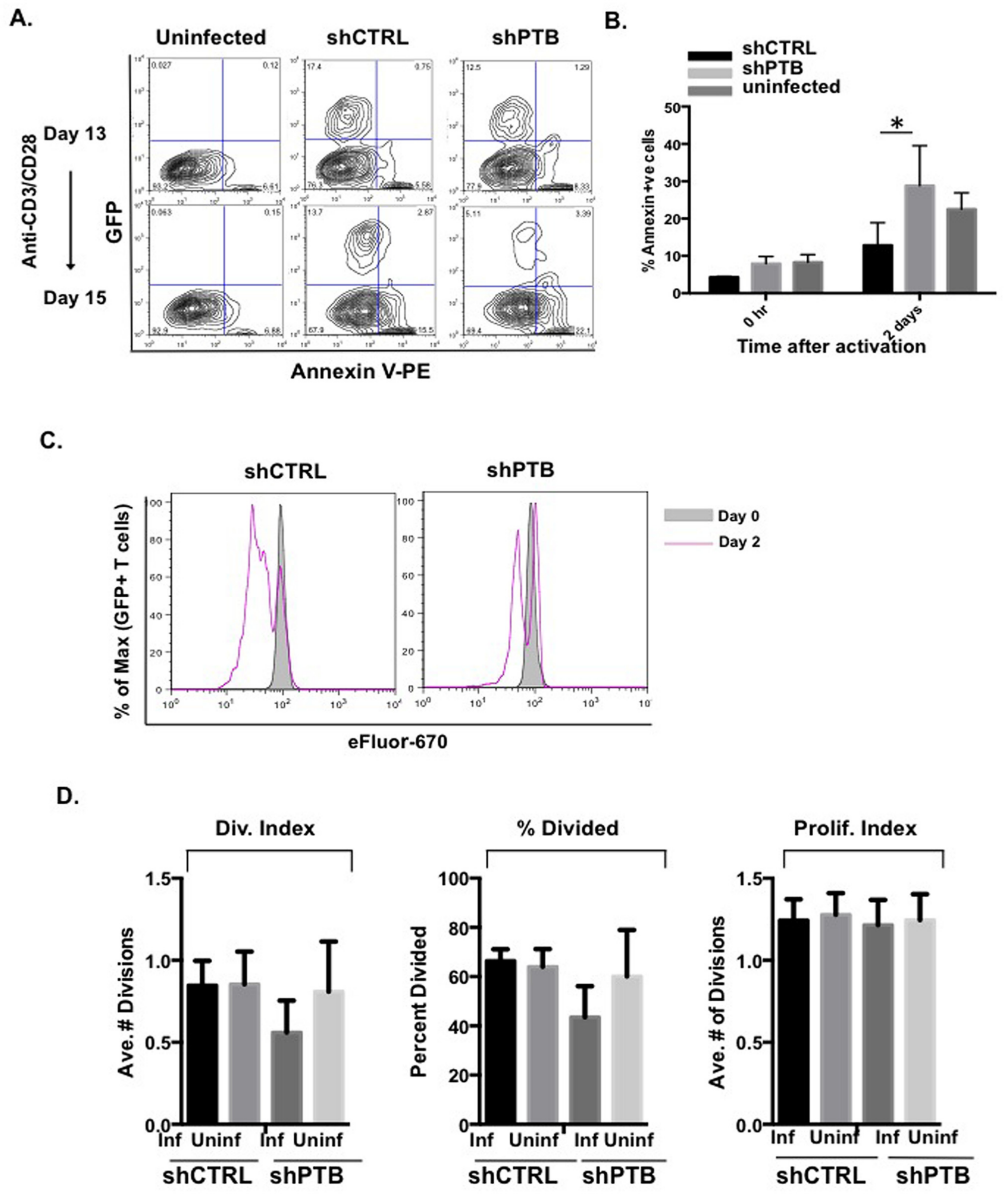
Reduced PTBP1 inhibits CD4 T cell Proliferation but not viability. (A) Analysis of cell viability in pLV-shCTRL or pLV-shPTB infected cells was carried out by staining 2×10^5^ cells with Annexin-V and analyzing GFPpos Annexin-Vpos cells prior to and after 48 h activation with anti-CD3 and anti-CD28 antibodies. Numbers shown are the percentage of PE-Anexin-Vpos cells in the total (GFPpos and GFPneg) population and comparisons were made between the pLV-shCTRL and pLV-shPTB infected cells as well as the infected and uninfected cells in the same population. (B) Compiled data from three independent experiments showing the extent of cell death in uninfected and pLV-shCTRL- and pLV-shPTB-infected T cells before and 48 h after stimulation with anti-CD3 and anti-CD28 beads. (C) 5 X 10^6^ pLV-shCTRL- and pLV-shPTB-infected T cells were incubated at day13 post-infection with 10 μM cell proliferation dye eFluor670 concurrently with the addition of anti-CD3/anti-CD28 conjugated beads for activation. Uninfected (GFPneg) and infected (GFPpos) populations were analyzed by flow cytometry at day 0 and day 2. (D) Division Index, Percent Divided and Proliferation Index were determined using FloJo software from three independent experiments (mean values (+/- SEM) with a *p≤0.05).

To examine whether PTBP1 protected against CD4 T cell death, Annexin V staining was carried out following a period of IL-2-mediated expansion, as well as two days of activation with anti-CD3/-CD28 mAb coated beads. As shown in Fig 2A and 2B, cells expressing reduced levels of PTBP1 showed no significant increase in cell death during the expansion phase of growth (see upper panels and “0 hr”). In contrast, there was a distinct increase in Annexin V positive cells following activation in the shPTB population (compare lower panels and day 2 results quantitated). Although, we cannot entirely exclude a role for PTBP1 in T cell survival during the expansion phase since cell death may occur at a threshold of PTBP1 that is lower than that present in our model, these findings indicate that lowering PTBP1 levels approximately 40% does not have a significant affect on the viability of IL-2-expanded CD4 T cells but is required for optimal survival in response to activation.

To further establish how PTBP1 affects proliferation, 10–12 day IL-2-expanded CD4 T cells were cultured in the absence of IL-2 for 1 day followed by culturing with anti-CD3/-CD28 mAb coated beads in the presence of the dye eFluor670 for two days (see representative experiment, Fig 2C). Populations of shCTRL- and shPTB-expressing cells were analyzed for the percentage of cells entering cell cycle, the number of cycles within the given time frame and the average rate of proliferation of individual cells. Our findings revealed that both the percentage of cells entering cell cycle and the average number of cycles were lower in shPTB-expressing cells. However, when the proliferation index was calculated for both the infected and uninfected populations there was no difference indicating that once a T cell had entered the cell cycle, events ensued at the same rate regardless of the level of PTB expression (Fig 2D). Therefore, based on our findings, the reduced number of shPTB-infected CD4 T cells in the IL-2 expanded population appears to be due to the impact of decreased PTBP1 expression on cell proliferation. However, following activation, both decreased proliferation and increased cell death were observed in cells expressing low PTBP1. Our findings are consistent with previous results showing that PTBP1 is required for proliferation [32, 34] and that decreasing PTBP1 levels in tumor cells can result in greatly enhanced cell death [35].

### Activation responses are compromised in cells expressing lower levels of PTBP1

**Fig 3:**
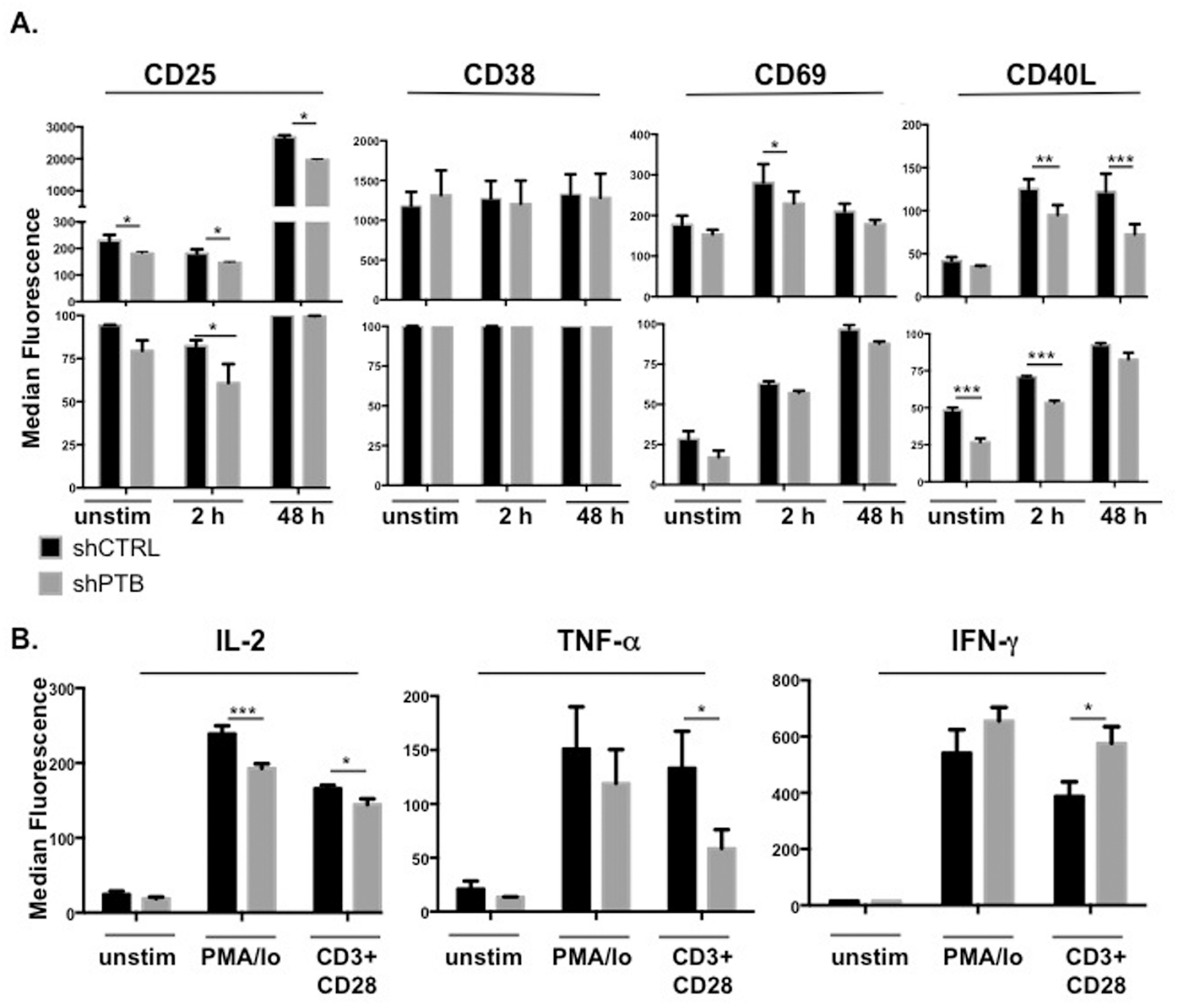
PTB is critical for expression of multiple activation markers. (A) pLV-shCTRL- or pLV-shPTB-infected CD4 T cells were expanded for 13 days and either left untreated or activated with anti-CD3/anti-CD28 beads for 2 h or 48 h. Following activation, cells were stained with antibodies to selected cell surface markers and analyzed for expression by flow cytometry. Results showing the median fluorescence intensity (top graph) and the percent positive (lower graph) are presented and the boxed bars indicate observed changes in absolute number of positive cells. (B) Expanded CD4 T cells were left untreated or activated with 1 ng/ml PMA and 1 μg/ml Ionomycin for 5 h or anti-CD3/anti-CD28 mAb-bound beads for 48 h and analyzed by intracellular staining with specific antibodies to IL-2, TNFα and IFNγ. Data shown represent the mean and SEM of three independent experiments with *p≤0.05, **p ≤ 0.01, and ***p≤0.005.

The influence of PTBP1 on the overall activation state of the CD4 T cells was further measured by analyzing expression of a number of activation responsive genes following CD3/CD28 activation for 2 or 48 h or stimulation for 5 h with PMA and ionomycin (PMA/Io). We found that similar to CD40L, decreased PTBP1 led to an overall reduction in CD25 and CD69 expression, corresponding to a drop in the absolute number of CD25 and CD69 positive cells (significance reached with CD69 only at 2 h due to the number of trials). In contrast, there was no change in the expression of CD38 either with respect to MFI or percent positive cells at both early and late times of activation (Fig 3A).

Expression levels of IL-2, TNFα and IFNγ were measured by intracellular staining following T cell activation with anti-CD3/anti-CD28 beads for 48 h as well as with PMA/Io for 5 h. We found that IL-2 and TNFα levels were reduced in shPTB-expressing cells, whereas IFNγ was increased under the same conditions (Fig 3B). Thus, CD40L, CD69, CD25, IL-2, TNFα, and IFNγ were found to be, in part, dependent on PTBP1 for optimal expression.

### IL-2, CD38 and CD40L are regulated through a program of activation-induced mRNA decay

**Fig 4:**
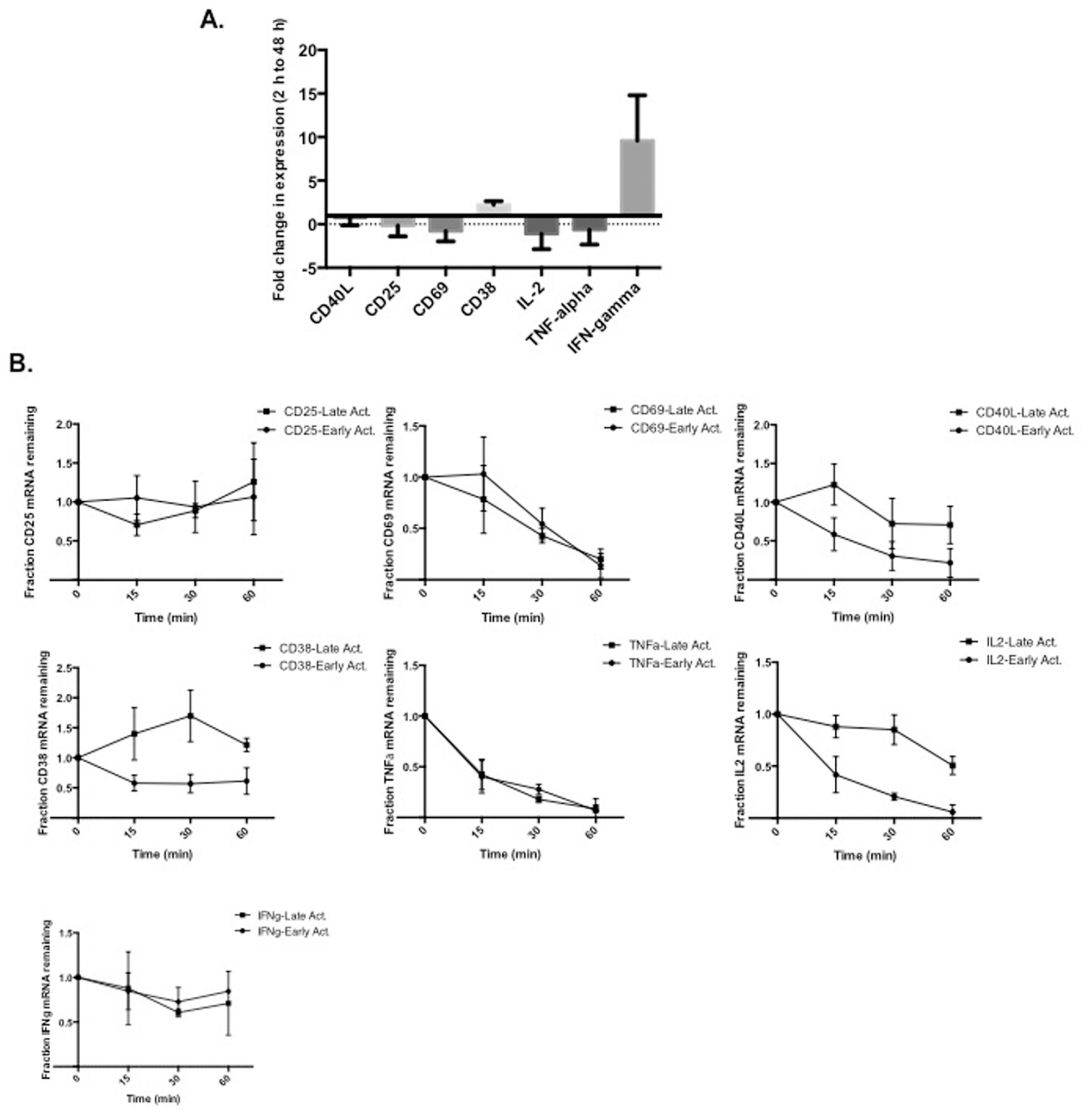
Steady-state RNA expression of different marker genes varies between early and late times of activation. (A) 5 X 10^6^ CD4 T cells were purified from total blood and cultured for 2 h or 48 h with anti-CD3 + anti-CD28 antibodies. Total RNA was reverse transcribed with random primers and analyzed using real time quantitative RT-qPCR. Shown is the fold change in expression between 2 h and 48 h of activation. (B) 2 h and 48 h stimulated CD4 T cells were incubated with DRB (50 μg/ml) for 15, 30, and 60 min. Analysis of RNA levels of the indicated targets was carried out following reverse transcription with poly(A) and RT-qPCR normalized to 18S RNA in each sample. Results represent the average and the SEM of three independent experiments.

To understand the associated mechanisms underlying PTB-mediated effects on the different activation responses, we first analyzed the overall expression patterns of the individual genes at early (2 h-6 h) and late (24 h-48 h) times of activation using real-time quantitative (q)PCR. We found that overall expression of CD25, CD69, IL-2 and TNFα decreased between the early and late time points whereas expression of both CD38 and IFNγ transcripts increased. In contrast, the expression level of CD40L was relatively unchanged between the early and late time points (Fig 4A).

To extend these findings and measure the decay rate of the different transcripts, expression was assessed in differentially activated uninfected CD4 T cells treated with the transcriptional elongation inhibitor DRB. In comparing the patterns for the individual activation genes we found only the CD40L, IL-2 and CD38 transcripts decayed with different kinetics at the early and late activation times. Specifically, IL-2 decayed with a *t*_*1/2*_ of less than 15 min at early time points and a *t*_*1/2*_ of approximately 60 min at late times. The CD38 transcript also decayed with a *t*_*1/2*_ of approximately 15 min at early times of activation and was found to be significantly stabilized at late activation time points (*t*_*1/2*_ > 60 min) (Fig 4B). Notably, the decay rates of CD25, CD69, TNFα and IFNγ were similar at both early and late time points.

**Fig 5:**
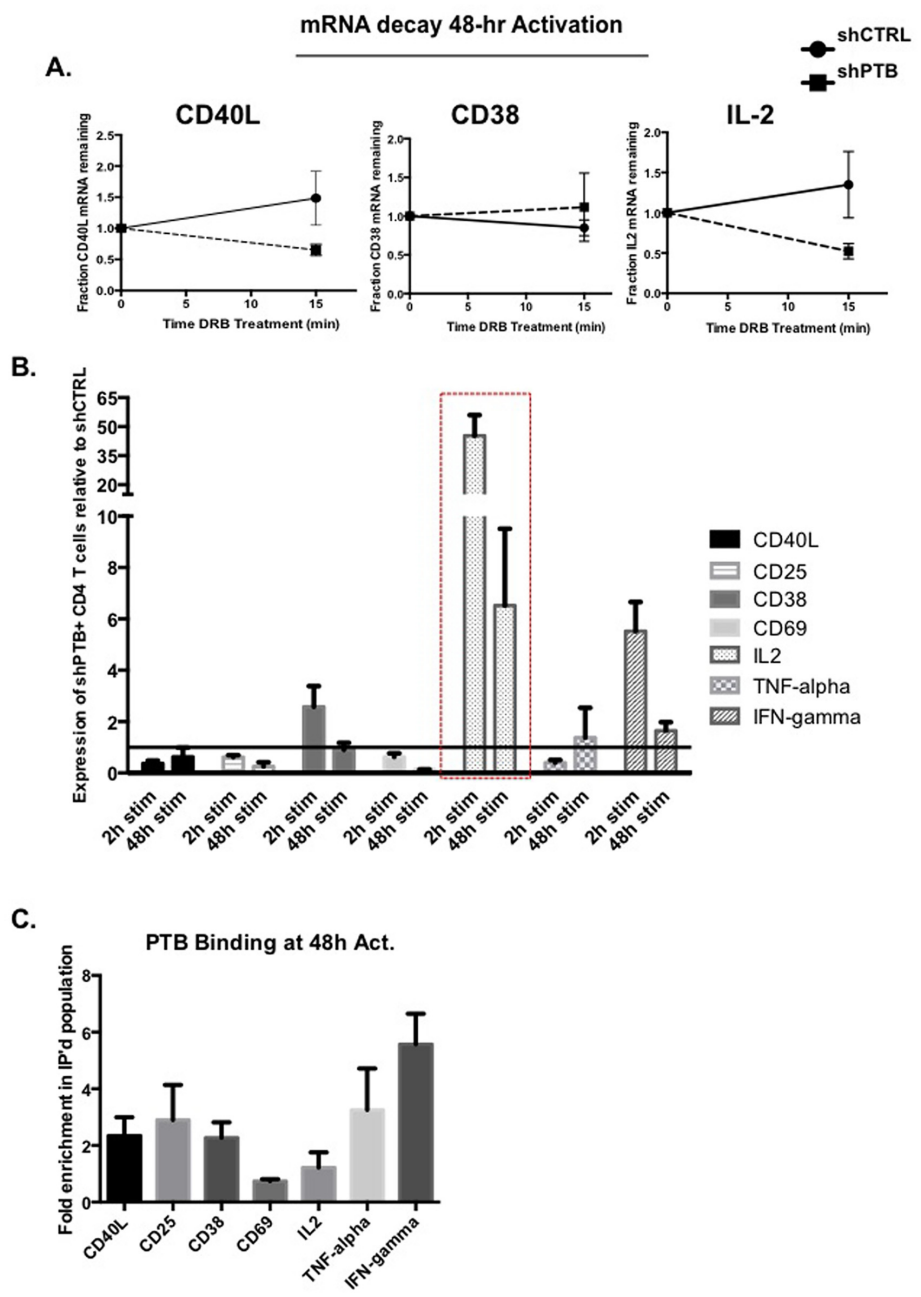
CD40L, CD38 and IL-2 messages are less stable in cells expressing shPTB. (A) Sorted GFPposCD4pos T cells expressing either pLV-shCTRL or pLV-shPTB were incubated with anti-CD3 +anti-CD28 antibodies for 48 h followed by treatment with 50 μg/ml DRB for 15 min. mRNA decay was analyzed by RT-qPCR normalizing against 18S as an internal control. (B) Total RNA was extracted from CD4 T cells expressing either shCTRL or shPTB and reverse transcribed using poly(dT) as a primer. Following incubation with anti-CD3 + anti-CD28 antibody-coated beads for 48 h RNA was quantified using real time qPCR using 18S as an internal control. Results are presented as the fold difference of values obtained in shPTB-expressing cells over those from shCTRL-expressing cells. (C) Cytoplasmic extracts from CD4 T cells activated for 48 h with anti-CD3 + anti-CD28 antibodies were immunoprecipitated with anti-PTBP1 antibodies. RNA was isolated and analyzed by RT-qPCR for enrichment of transcripts in the bound fraction relative to the transcript representation in total cytoplasm.

We next asked whether PTBP1 had a role in the activation-induced stabilization of the CD38 and IL-2 transcripts at late times of activation. For these experiments GFP sorted, shPTB- and shCTRL-infected primary CD4 T cells were activated with anti-CD3/-CD28 mAb for 48 h and the transcriptional inhibitor DRB was added during the last 15 min of the 48 h culture. Total RNA was isolated, reversed transcribed using poly(A) primer and analyzed by qPCR. A comparison of mRNA levels at time 0 (arbitrarily set to 1) to the 15 min time point revealed that the decay of the CD38 transcript was not affected by decreased PTBP1 whereas similar to CD40L, the IL-2 transcript was less stable in cells expressing shPTB (Fig 5A).

Total RNA expression of the seven activation genes was further analyzed in 2 h- and 48 h-stimulated and FACS sorted GFPposCD4pos T cells. In comparing transcripts levels between the two infected populations, we found that the level of CD40L mRNA was reduced in cells expressing shPTB at both 2 h and 48 h supporting our previous results showing decreased CD40L protein expression at these time points [22]. Additionally, TNFα, CD25 and CD69 mRNA levels were greatly reduced following activation (Fig 5B). We also found that PTBP1 acted as a negative regulator of IFNγ expression since there was an increase in IFNγ transcripts at both early and late times of activation in cells expressing shPTB. These findings closely aligned with our FACS data showing PTBP1 can act as a positive or negative factor in regulating expression of specific activation genes (Figs 3 and 4). A surprising result was the high level of IL-2 mRNA in shPTB-expressing cells at both activation time points. This was unexpected given the fact that IL-2 protein levels decreased and the mRNA was found to be less stable in cells with downregulated PTBP1. This finding suggests that PTBP1 has additional functions in IL-2 gene regulation that could encompass, for example, the transport and/or translation of IL-2 mRNA.

To determine whether the effect of PTBP1 on expression of the different genes could be due to direct interactions, cytoplasmic extracts were prepared from CD4 T cells stimulated for 48 h with anti-CD3 and anti-CD28 antibodies and immunoprecipitated with anti-PTBP1. As expected we found that the CD40L transcript was enriched in the immunoprecipitated fraction approximately 2-fold (Fig 5C). This level is very close to the 1.8-fold enrichment obtained using immunoprecipitated PTBP1-bound transcripts and microarray analysis (data not shown). Furthermore, we found that CD25, CD38, TNFα, and IFNγ transcripts were also enriched in the bound fraction relative to the total extract. In contrast, we saw little to no enrichment of the IL-2 and CD69 transcripts (Fig 5C). We conclude that PTBP1 is critical for the optimal expression of multiple activation genes, which occurs in some cases through PTBP1 binding to specific transcripts and in other cases as a consequence of indirect interactions.

### PTBP1 regulates activation in T cells through altered signaling

**Fig 6:**
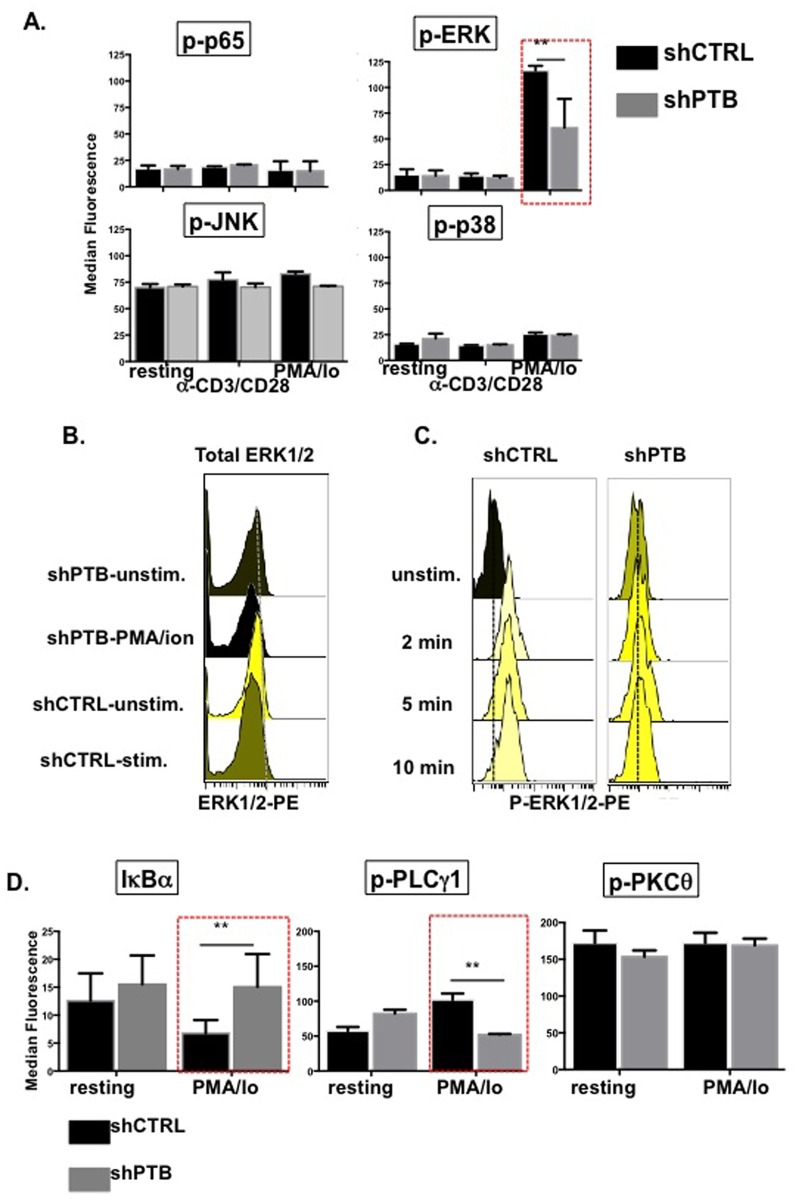
PTBP1 regulates ERK1/2 and NF-κB signaling. (A) CD4 T cells infected with either pLV-shCTRL or pLV-shPTB lentivirus were activated with either anti-CD3/anti-CD28 beads or 1 ng/ml PMA and 1 μg/ml Io between 2 and 20 min (depending on the optimal response of individual signals). Phosphflow analysis was carried out by fixing cells in paraformaldehyde and permeablizing them for intracellular staining with antibodies to the indicated targets. (B) Histograms showing total (left panel) and phosphorylated ERK1/2 (right panel) in PMA/Io stimulated GFPposCD4pos T cells. (C) Histograms showing ERK1/2 signaling in cells expressing shCTRL and shPTB over 10 min of stimulation with PMA/ionomycin. (D) CD4 T cells infected with either pLV-shCTRL or pLV-shPTB lentivirus were activated with 1 ng/ml PMA and 1 μg/ml Ionomycin for 10 min and analyzed for PLCγ1, PKCθ, and IκBα activity (mean values (+/- SEM) with a *p≤0.05).

Because of the broad impact that PTBP1 has on CD4 T cell activation we next focused on identifying signaling pathways that were dependent on PTBP1 expression. To this end, infected CD4 T cells were expanded, rested and activated with either anti-CD3/anti-CD28 beads or PMA/Io and analyzed for pathway activation using anti-phospho-specific antibodies and flow cytometry. In resting CD4 T cells, there were very low levels of p65, ERK, and p38 activity and higher basal levels of JNK (Fig 6A). Following stimulation with anti-CD3/anti-CD28 beads the levels of JNK, p38, p65, and ERK1/2 remained unchanged relative to basal levels. However, when cells were exposed to PMA/Io we observed a significant loss of ERK1/2 activity in shPTB-expressing cells. To ensure that we were observing a decrease in ERK1/2 activity and not just a decrease in overall protein level, intracellular staining was carried out using antibodies against total ERK1/2 and no differences were observed (Fig 6B). We also observed no differences in the overall expression of JNK, p38, p65 and PLCγ1 between the two cell populations (S2 Fig). To determine whether decreased ERK1/2 activity reflected a delay rather than a decrease in activation, ERK1/2 activity was monitored in shCTRL- and shPTB-expressing cells over a time course of stimulation. Because the response of these cells was lower at each time point, we conclude that the difference in ERK1/2 activity reflected an overall decrease and not just a delay in response of cells expressing lower overall levels of PTBP1 (Fig 6C).

Due to the fact that p65 activation was not observed under our conditions of stimulation, we further analyzed the NF-κB pathway by assessing the degradation of IκBα an inhibitor of NF-κB1 translocation into the nucleus. As shown in Fig 6D, Tcells with reduced PTBP1 failed to degrade IκBα in response to activation indicating impaired NF-κB signaling in these cells. We further analyzed the phosphorylation of PLCγ1 and PKC-θ signaling events that occur upstream of ERK1/2 and NF-κB [36]. Surprisingly, we found no difference in PKC-θ activity in the two populations, however there was a distinct decrease in PLCγ1 signaling in shPTB-expressing cells. This finding suggests that PTBP1-dependent changes occur very early in T cell signaling (Fig 6D). Thus, downregulating PTBP1 results in decreased PLCγ1/ERK1/2, and IκBα within an early window of activation that precedes changes in cytokine expression and cell division.

## Discussion

T cell activation is defined by a cascade of transcriptional, post-transcriptional and translational events that lead to the timely differentiation of functional T cell subsets expressing distinct cytokine and functional profiles [37, 38]. Although much is known regarding the transcriptional regulation and signal transduction underlying T cell activation, the functional outcomes of posttranscriptional mechanisms are less well defined. The importance of unraveling the relationship between RNA processes and activation is supported by the recognized significance of microRNA in controlling gene expression [39, 40] as well as the finding that greater than 50% of induced expression changes in T cells occur as a consequence of regulated mRNA decay [41]. In this work, we provide strong evidence to support a critical role for PTBP1 in a wide range of activation events. Importantly, PTBP1-mediated gene regulation is shown to occur at multiple levels including mRNA decay, and the final result is primarily an attenuation of activation responses.

Importantly, all our observations were made with PTBP1 expression being decreased approximately 40% and therefore our overall observations may greatly underestimate the significance of PTBP1 in multiple facets of T cell activation. The observation that PTBP1 is critical for proliferation is consistent with previous studies showing that embryonic stem cells lacking PTBP1 have a block in G2/M progression that corresponds to defective CDK11^p58^ IRES-dependent translation [32, 34]. Also, knockdown of PTBP1 in a variety of tumors and in multiple transformed cell lines where PTBP1 is overexpressed results in impairment of both growth and invasive potential of specific cell lines [42–44]. It is noteworthy that we observed PTBP1-associated proliferation defects in the IL-2-dependent expansion phase as well as during activation with either anti-CD3/-CD28 mAbs or with PMA/Io (data not shown). This suggests that the role PTBP1 plays in cell division is not dependent on a specific surface molecule and more likely linked to a common event downstream of ligand engagement. This possibility is also compatible with the effect of PTBP1 on ES and tumor cell proliferation [32, 34, 45].

The observed decreases in ERK1/2 and NF**-**κB activity in cells expressing shPTB was particularly evident in cells stimulated with PMA/Io compared to anti-CD3 and anti-CD28 activation. This finding strongly aligns with previous reports showing that MAPK signaling is more potent with PMA/Io compared to activation through the TCR and co-stimulatory molecules [46, 47]. Our observation of decreased ERK1/2 activity with corresponding changes in proliferation and STAT5 activation in shPTB-expressing T cells supports other studies demonstrating a requirement for ERK1/2 signaling at multiple stages of the cell cycle (reviewed in [48]). For example, ERK1/2 phosphorylates the protein phosphatase CDC25A, which dephosphorylates multiple cell cycle proteins necessary for G1 progression and G1/S cell cycle transition [49]. ERK1/2 also regulates cyclin D1 transcription through phosphorylation of the negative transcriptional co-repressor Tob [50, 51] and acts to control the expression of cyclin D1 at the posttranscriptional level [52–55]. Finally, ERK1/2 positively regulates the cell cycle by increasing the availability of nutrients [56], stimulating the cyclin-dependent kinase (CDK) complexes [57], and preventing cell death [58, 59]. ERK1/2 also has been shown to target c-Myc resulting in increased c-Myc stabilization [60].

Loss of PTBP1-dependent ERK1/2 signaling may have cellular consequences separate from proliferation. PTBP1 has been shown to be critical for the generation of pyruvate kinase isoforms and associated aerobic glycolysis in cancer cells (known as the “Warburg effect”) [25, 61]. Gene silencing of PTBP1 in human colon cancer cell lines resulted in an increase in the PKM1/PKM2 ratio and induced apoptosis and/or autophagy of the targeted cells [35]. The fact that T cells also utilize glycolysis during activation [62] makes it likely that PTBP1 is required for glycogen utilization in proliferating T cells and a drop in levels could result in cell death similar to what we observed with activation. Notably, enhanced glucose uptake and glycolysis by activated T cells was reported to be dependent on ERK1/2 signaling potentially through the regulation of hexokinase expression and activity [63].

We found PTBP1 to be critical for optimal CD25 and CD69 mRNA and protein expression. Because there was no observed difference in the decay pattern of these transcripts in GFPposCD4pos cells, we conclude that PTBP1 positively regulates the transcription of CD25 and CD69. The fact that CD25 and CD69 transcription requires the AP-1 and NF**-**κB transcription factors [64, 65] which require PLCγ1 and ERK1/2 activation may explain why reduced levels of CD25 and CD69 transcripts are observed in shPTB-expressing cells. Decreased TNFα transcripts in these cells at 2 h of activation may also be explained by lowered signaling responses in the ERK1/2 and NF**-**κB pathways [66]. In contrast, PTBP1 appears to negative regulate IFNγ expression as shown by increased RNA and protein in CD4 T cells expressing shPTB. This result is surprising given that IFNγ also requires AP-1 activity for transcription however increased expression of IFNγ is also observed in mice lacking the Roquin and Roquin-2 genes that encode RNA binding proteins essential for the degradation of several mRNAs involved in T cell activation [67].

A comparison of mRNA expression for late activated CD4 T cells revealed only activation-induced changes in mRNA stability for the CD40L, CD38 and IL-2 transcripts. The processes underlying CD40L and IL-2 message stabilization have been extensively studied and appear to be mechanistically distinct with IL-2 regulated by both ARE-dependent and independent pathways [68, 69]. The observed change in the IL-2 mRNA at 48 h of activation is consistent with shPTB-expressing cells being less fully activated and therefore the decay is more similar to the rapid decay found in 2 h stimulated cells. One intriguing interpretation of our data is that decreased PTBP1 and associated ERK1/2 signaling initiates a cascade of events that results in cells entering an anergic-like state [70]. The increase in IL-2 mRNA with decreased levels of the cytokine in shPTB-expressing T cells is consistent with previous work showing that the lack of cytokine expression in an in vivo model of T cell anergy was associated with a block in mRNA translation [71].

Overall, our studies reveal a critical role for PTBP1 in regulating diverse processes required for optimal T cell activation. It is intriguing to speculate that a subset of PTBP1-mediated defects may be potentially due to a loss of PTBP1-controlled microRNA function. This possibility is supported by recent findings that PTBP1 interacts with microRNAs to regulate the expression of a number of transcripts in diverse cell types [35, 72–76].

## Supporting Information

S1 Fig CD3 and CD28 expression is similar on primary human CD4 T cells infected with shCTRL and shPTB. (TIF)

S2 Fig Steady-state levels of signaling molecules are unchanged in activated CD4 T cells expressing shCTRL or shPTB. (TIF)

S1 Table Sequences of primers used in real time PCR. (DOCX)
