# Supplementary figures and images for: The RNA-Binding Protein, Polypyrimidine Tract-Binding Protein 1 (PTBP1) Is a Key Regulator of CD4 T Cell Activation
Source: PLoS One. 2016 Aug 11;11(8):e0158708. doi: 10.1371/journal.pone.0158708 (PMC4981342; doi:10.1371/journal.pone.0158708)

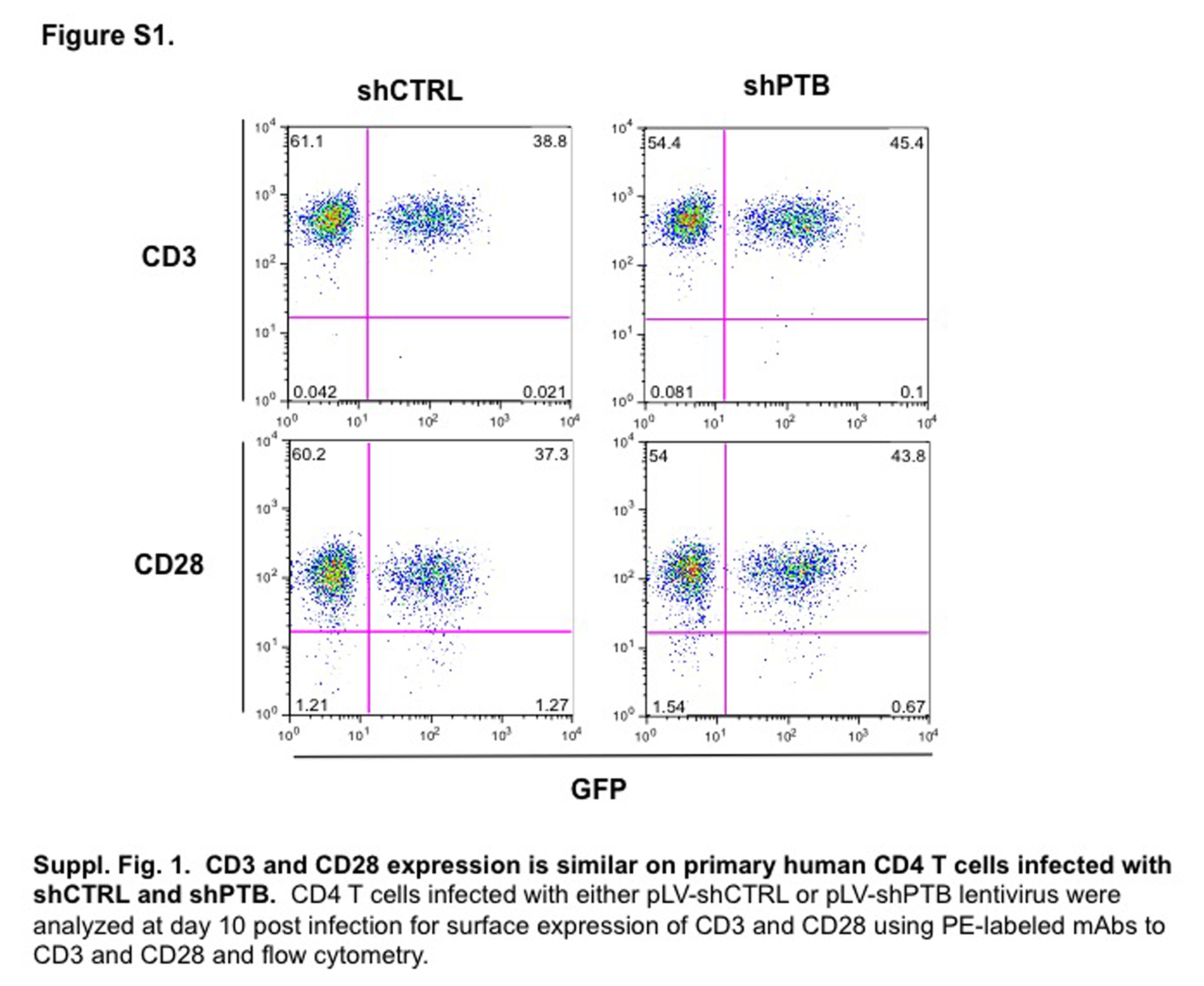

Supplement: S1 Fig — (TIF) [file pone.0158708.s001.tif]

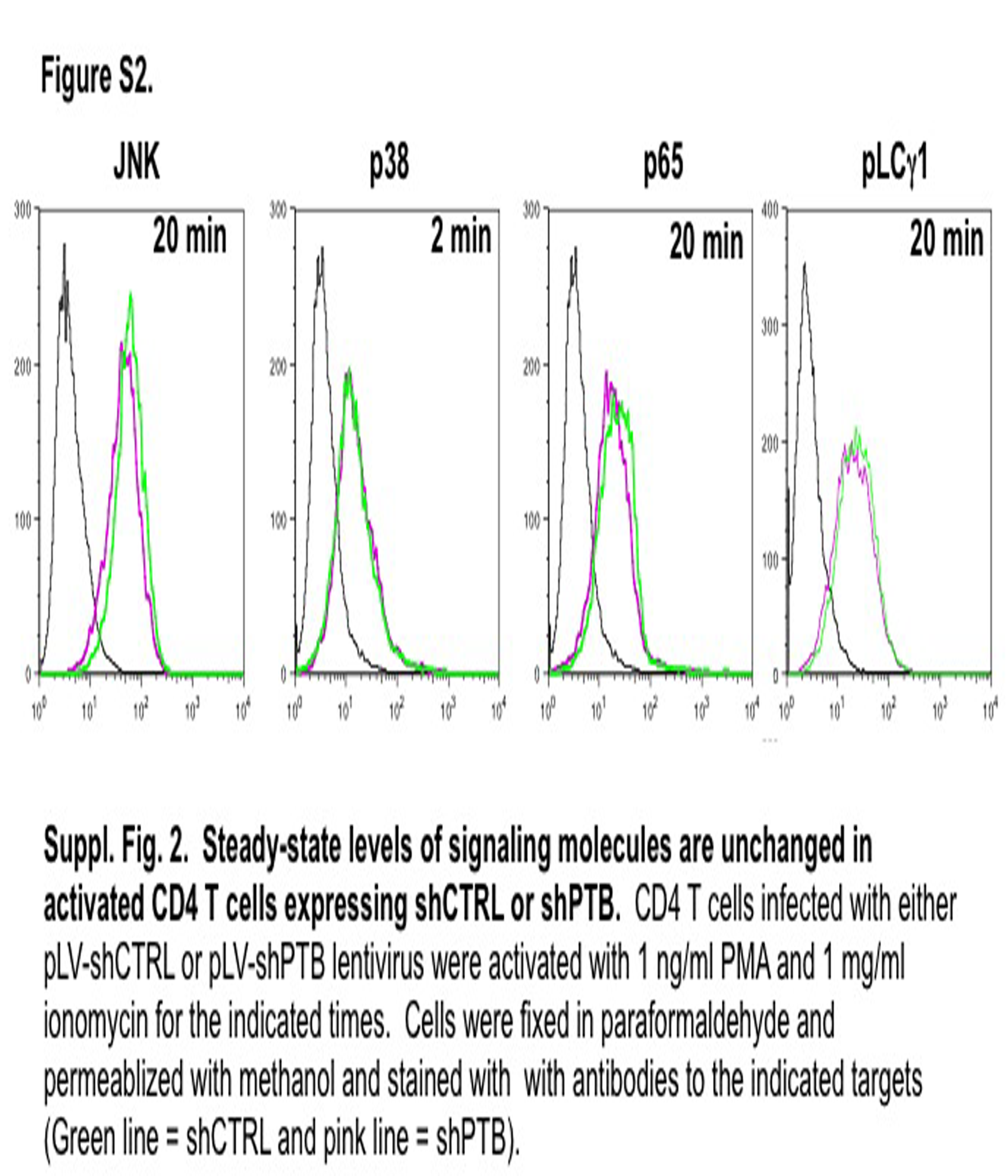

Supplement: S2 Fig — (TIF) [file pone.0158708.s002.tif]
