# Supplementary material for: The RNA-Binding Protein, Polypyrimidine Tract-Binding Protein 1 (PTBP1) Is a Key Regulator of CD4 T Cell Activation
Source: PLoS One. 2016 Aug 11;11(8):e0158708. doi: 10.1371/journal.pone.0158708 (PMC4981342; doi:10.1371/journal.pone.0158708)
Supplement: S1 Table — (DOCX) [file pone.0158708.s003.docx]

| CD40L | \| hCD40L.10Fwd \|  \| \| --- \| --- \| | ACATACAACCAAACTTCTCCCCG |
| --- | --- | --- | --- | --- |
|  | hCD40L.128-Rev | GCAAAAAGTGCTGACCCAATCA |
| CD25 | hCD25.166.Fwd | CGCAGAATAAAAAGCGGGTCA |
|  | hCD25.281.Rev | ACTTGTTTCGTTGTGTTCCGA |
| CD69 | huCD69.200.Fwd | ATTGTCCAGGCCAATACACATT |
|  | huCD69.418.Rev | CCTCTCTACCTGCGTATCGTTTT |
| CD38 | huCD38.240.Fwd | AGACTGCCAAAGTGTATGGGA |
|  | huCD38.357.Rev | GCAAGGTACGGTCTGAGTTCC |
| IL-2 | hIL2.14.Fwd | AACTCCTGTCTTGCATTGCAC |
|  | hIL2.106.Rev | GCTCCAGTTGTAGCTGTGTTT |
| IFN-γ | hIFNG.364.Fwd | TCGGTAACTGACTTGAATGTCCA |
|  | hIFNG.456.Rev | TCGCTTCCCTGTTTTAGCTGC |
| TNF-α | hTNFa.274.Fwd | CAGCCTCTTCTCCTTCCT |
|  | hTNFa.396.Rev | GCCAGAGGGCTGATTAGAGA |
| 18S | 18S.Fwd | CTCAACACGGGAAACCTCAC |
|  | 18S.Rev | CGCTCCACCAACTAAGAACG |
| β-actin | hbactin.75.FWD | CCAGCTCACCATGGATGATG |
|  | hbactin.131.Rev | ATGCCGGAGCCGTTGTC |

**Table S1.** Sequences of primers used in real time PCR
